# Serial patent litigation: an emerging strategy to delay entry of generic competition

**DOI:** 10.1093/haschl/qxaf240

**Published:** 2025-12-17

**Authors:** Timothy Bonis, Aaron S Kesselheim, Sean Tu

**Affiliations:** Dartmouth College, Hanover, NH 03755, United States; Program On Regulation, Therapeutics, And Law (PORTAL), Division of Pharmacoepidemiology and Pharmacoeconomics, Department of Medicine, Brigham and Women's Hospital, Boston, MA 02120, United States; Program On Regulation, Therapeutics, And Law (PORTAL), Division of Pharmacoepidemiology and Pharmacoeconomics, Department of Medicine, Brigham and Women's Hospital, Boston, MA 02120, United States; Harvard Medical School, Boston, MA 02115, United States; Program On Regulation, Therapeutics, And Law (PORTAL), Division of Pharmacoepidemiology and Pharmacoeconomics, Department of Medicine, Brigham and Women's Hospital, Boston, MA 02120, United States; University of Alabama School of Law, Tuscaloosa, AL 35487, United States

**Keywords:** generic drug, litigation, delay, patents

## Abstract

The Hatch–Waxman Act of 1984 was designed to accelerate generic drug entry by establishing a framework for resolving patent disputes between brand-name and generic manufacturers. While the Act has facilitated competition and expanded the availability of affordable medicines, brand-name firms have increasingly exploited its procedural structure to delay or deter generic competition through “serial litigation.” This strategy involves filing successive, questionable lawsuits, often based on non-innovative continuation patents. Even if the brand ultimately loses, the delays and litigation costs can discourage generic firms from entering the market or compel them to settle on terms that undermine patients’ timely access to affordable generics. In the case of Astellas's overactive bladder drug mirabegron (Myrbetriq), after an initial Hatch–Waxman case settled in 2020 with generic entry expected in 2024, Astellas pursued 4 additional lawsuits, each built on new but substantively indistinguishable patents. These tactics have delayed broad competition, leaving only 2 firms to launch in 2024 under the threat of massive damages. Similar patterns are observed with other drugs, including bimatoprost (Latisse), aflibercept (Eylea), and tasimelteon (Hetlioz).

A core tenet of the U.S. legal system is that parties should get only 1 opportunity to litigate and appeal a case. But this so-called one bite at the apple principle, which is vital to protecting defendants from the burden of repeat litigation, is increasingly being undermined by brand-name drug manufacturers seeking to prevent generic and biosimilar competition.

The Hatch–Waxman Act of 1984 formalized the current process through which generic drugs reach the U.S. market. The Hatch–Waxman Act led to increased competition in the drug market and more affordable medications. Under the Hatch–Waxman Act, brand-name manufacturers must list the key patents protecting their products with the U.S. Food and Drug Administration (FDA). Typically, generics manufacturers launch their products only after challenging the validity or relevance of these patents. These challenges lead the brand-name manufacturer to initiate litigation, which, once resolved, clears the way for generic availability.

The litigation framework established by the Hatch–Waxman Act was intended to simplify generic entry. Recently, however, brand-name firms have been using the litigation procedure to delay or deter generic and biosimilar competition by filing successive, nearly identical patent infringement lawsuits. We call these “serial litigations.” Even if the brand-name manufacturer loses one litigation, it can sue again using a technically new, but in reality nearly identical, patent. These additional lawsuits create procedural delays and increase legal costs. Because Hatch–Waxman cases cost generic companies $6.2 million on average,^1^ litigation costs may exceed short-term expected profits. Thus, generic manufacturers may choose to stay off the U.S. market or settle in ways that undermine patients’ timely access to affordable generics.

This serial litigation strategy is exemplified by the blockbuster overactive bladder drug mirabegron (Myrbetriq; Astellas), a chronic medication that has an estimated average annual net price of around $977. Astellas initiated 5 successive rounds of lawsuits against prospective generic entrants starting in 2016. The first 2016 lawsuit was a routine Hatch–Waxman litigation; Astellas sued 9 generic manufacturers seeking to produce mirabegron. This case settled in April 2020, with Astellas seemingly allowing generic availability in April 2024.

Then, in November 2020, Astellas sued all 9 generics again, beginning the first of 4 additional “serial” lawsuits. The 2020 case used a new patent that Astellas had obtained during its first lawsuit. The 9 generics won initially (courts invalidated the patent), but Astellas appealed, and the prospect of continued litigation compelled all but 3 potential generic manufacturers (Lupin, Zydus, Sandoz) to settle a second time, reaching confidential agreements and remaining off the market.^2^

In June 2023, Astellas received another patent and filed a third lawsuit, targeting the 3 firms seeking to market generic versions in the United States that had not yet settled the prior case. Although a court doubted the patent's validity and denied Astellas's initial motion, the case has continued and prompted Sandoz to resettle.^3^

In 2024, Lupin and Zydus, the only generic firms left, launched their products at-risk of a patent infringement lawsuit, chancing liability of hundreds of millions if Astellas won either of the ongoing lawsuits. Astellas responded with 2 *more* lawsuits based on 2 new patents.^4^ Tellingly, these most recent patents are nearly identical in substance to the patents from the second and third lawsuits (see Box 1) and are legally designated as “continuation patents” or “children” of those earlier patents. The fact that Astellas has pursued “child” patents demonstrates that its approach relies on procedural gamesmanship rather than substantive patent rights. While U.S. patent law permits “child” patents to be granted, they are limited in other countries.^5^

Box 1. Mirabegron litigated patent claims.

**Table:** 

Litigation 2 (2017)	Litigation 4 (2024)
Original Patent (10 842 780)	Continuation Patent (12,059,409)
“A pharmaceutical composition, comprising [mirabegron]…in a sustained release hydrogel-forming formulation comprising a hydrogel-forming polymer having an average molecular weight of 100,000 to 8,000,000…”	“A tablet, comprising [mirabegron]…in a sustained release hydrogel-forming formulation comprising a hydrogel-forming polymer having an average molecular weight of 200,000 to 7,000,000…”
“…wherein the hydrogel-forming polymer is at least one compound selected from the group consisting of polyethylene oxide, hydroxypropyl methylcellulose, hydroxypropyl cellulose, carboxymethyl cellulose sodium, hydroxyethyl cellulose, and a carboxyvinyl polymer.”	“…wherein the hydrogel-forming polymer is polyethylene oxide.”
“…wherein the additive is at least one selected from the group consisting of polyethylene glycol, polyvinylpyrrolidone, D-mannitol, D-sorbitol, xylitol, lactose, sucrose…” (20 additives listed)	“…wherein the additive is polyethylene glycol”

**Table:** 

Litigation 3 (2020)	Litigation 5 (2024)
Original Patent (11,707,451)	Continuation Patent (12,097,189)
“A method for treating overactive bladder… the method comprising administering orally to a subject in need thereof a tablet comprising 10 mg to 200 mg of [mirabegron] in a sustained release formulation…selected from the group consisting of a sustained release hydrogel-forming formulation, a multi-layered formulation [], a gel formulation [], an osmotic pump type formulation [], a matrix formulation [], a modified release formulation [], and a matrix formulation [].”	“A method for treating overactive bladder…the method comprising administering orally to a subject in need thereof a tablet comprising **25 **mg of [mirabegron] in a sustained release hydrogel-forming formulation”
“wherein the reduced food effect is compared to that after oral administration of an immediate release formulation comprising [mirabegron].”	“wherein the reduced food effect is compared to that after oral administration of an immediate release formulation comprising [mirabegron] and is a difference…of C_max_ of 10% or more…”
Examples of claims from the patents asserted in mirabegron lawsuits 2 to 5. The patents in litigations 4 and 5 have no substantial differences from the patents in litigations 2 and 3.	

By using the litigation process in this way, Astellas has impeded generic entry regardless of whether it wins the underlying litigation. Instead of a competitive market served by potentially up to a dozen manufacturers in April 2024, as established in the first settlement, generic mirabegron became available only because 2 firms launched at-risk. The litigation compelled other competitors to delay or abandon market entry, putting less pressure on prices. This cycle is far from over, as Astellas is pursuing 2 additional patents, and the current litigation is unresolved.

Multiple brand-name manufacturers are employing serial litigations (Table 1). In the case of bimatoprost (Latisse; Allergan), generics could have entered in 2014 following their initial legal victory. Instead, Allergan filed 2 nearly identical lawsuits, delaying entry until 2016, then sued again in 2017.^6^ Litigation over aflibercept (Eylea; Regeneron) and tasimelteon (Hetlioz; Vanda) has followed this pattern.

**Table 1.** Examples of serial litigations and potential generic delay.

Drug	Number of lawsuits	Duration of litigation	Potential generic delay (excluding at-risk launches)
Mirabegron (Myrbetriq; Astellas)	5	9 years, 2 months (10/2016-present)	5 years, 10 months (5/2024, original settled date → 3/2030, expiration of last patent in last case)
Aflibercept (Eylea; Regeneron)	2	2 years (12/2023-present)	2 years, 7 months (11/2024, first litigation won by generics → 6/2027 expiration of last patent in last case)
Bimatoprost (Latisse; Allergan)	4	15 years, 2 months (9/2010-present)	6 years, 7 months (6/2014, first litigation won by generics → 1/2021, expiration of last patent in last case)
Tasimelteon (Hetlioz; Vanda)	3	7 years, 10 months (2/2018-present)	12 years, 8 months (12/2022, first litigation won by generics → 8/2035, expiration of last patent in last case)

Serial litigation is poised to become more common as the conditions that support this strategy have grown over the past 2 decades. First, the number of patents associated with each drug listed with the FDA tripled between 1980 and 2019, expanding the raw material needed for repeat lawsuits.^7^

Additionally, a growing share (now nearly half) of FDA-listed patents are the child patents frequently employed in serial litigation.^8,9^ These patents are expressly non-innovative; patentees admit during the application process that they are derivatives of prior patents. Because every additional patent can support a new infringement claim, acquiring child patents permits brand manufacturers to mount a series of follow-on lawsuits despite the absence of new underlying issues.

Large patent estates with many child patents are preconditions for serial litigation. Given that both are on the rise, we are likely only beginning to observe the potential for this tactic to impede the timely availability of affordable generic medicines. Indeed, courts have observed its growing prevalence. In 2024, Judge Andrews of the District of Delaware, who has adjudicated the most Hatch–Waxman cases since 2016, remarked: “Given what I have seen in recent years, I am not as confident that a branded company, given the option of repeat litigation… would be deterred from that litigation simply because its chance of victory was poor.”^10^

Legal, administrative, and legislative reforms could help prevent serial litigation. The law's collateral estoppel doctrine stops the reuse of arguments that emerged and were addressed in a previous case. A stronger collateral estoppel doctrine would give practical effect to the 1 bite at the apple principle by preventing brand-name manufacturers from repeating infringement claims in multiple litigations. However, collateral estoppel rules are judge-decided and inconsistent, and many courts are reluctant to apply the doctrine. Federal standards could help clarify how and when to apply collateral estoppel in serial drug patent litigation cases.

Alternatively, courts could require brand-name manufacturers to surrender profits accrued by delaying generics through meritless serial litigation, applying criteria already established to distinguish litigation that is unreasonable, abusive, or in bad faith. An Israeli court used this solution against a brand-name firm found to have improperly delayed generic entry. Canadian courts have also granted lost profits revenue due to unjust delays.

An administrative solution could be based on a previously proposed U.S. Patent and Trademark Office rule that would have made unenforceable all the child patents of an invalidated patent. Similarly, the bipartisan Eliminating Thickets to Increase Competition Act, proposed in 2025, would limit firms’ ability to assert multiple child patents.

Serial litigation inflates costs for generic and biosimilar manufacturers, blocking timely market entry and forcing patients and the health care system to bear higher drug prices. Preventing this type of gamesmanship is essential to ensuring timely access to generics and biosimilars, driving down drug prices, and ultimately improving patient access and health outcomes.

## Supplementary Material

qxaf240_Supplementary_Data
